# The Actin Cytoskeleton as a Barrier to Virus Infection of Polarized Epithelial Cells

**DOI:** 10.3390/v3122462

**Published:** 2011-12-21

**Authors:** Elizabeth Delorme-Axford, Carolyn B. Coyne

**Affiliations:** Department of Microbiology and Molecular Genetics, University of Pittsburgh, 518 Bridgeside Point II, 450 Technology Drive, Pittsburgh, PA 15219, USA

**Keywords:** polarized epithelium, actin cytoskeleton, tight junctions, virus entry

## Abstract

Many diverse viruses target a polarized epithelial monolayer during host invasion. The polarized epithelium is adept at restricting the movement of solutes, ions, macromolecules, and pathogens across the mucosa. This regulation can be attributed to the presence of a junctional complex between adjacent cells and to an intricate network of actin filaments that provides support to the subapical membrane and stabilizes intercellular junctions. It is therefore not surprising that many viruses have evolved highly varied strategies to dissolve or modulate the cortical actin meshwork to promote infection of polarized cells. In this review, we will discuss the cell biological properties of the actin cytoskeleton in polarized epithelial cells and review the known mechanisms utilized by viral pathogens to manipulate this system in order to facilitate their infection.

## 1. Introduction

**Figure 1:**
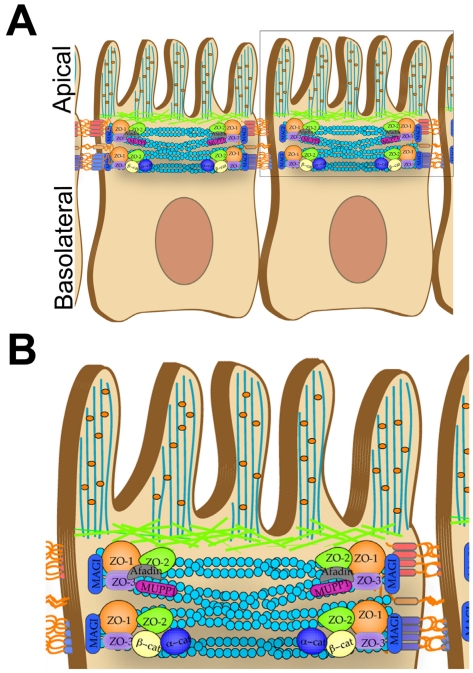
Model of Polarized Epithelial Cells. (**A**) Polarized intestinal epithelial cells (IECs) lining the gastrointestinal (GI) tract have distinct apical and basolateral surface domains. The apical domains are intimately associated with the actin cytoskeletal network. Microvilli localize to the apical surface and are anchored by interactions with actin-binding proteins ezrin, radixin, and moesin (ERM). (**B**) The highlighted area of the apical domain demonstrates the close interactions between actin and junctional complex proteins [ZO-1, ZO-2, ZO-3, Afadin, multi-PDZ domain protein 1 (MUPP1), MAGI, α- and β-catenin].

Several families of viruses target a polarized epithelial monolayer during their life cycles. Airway pathogens such as rhinovirus, respiratory syncytial virus, and influenza virus invade via the polarized epithelia lining the airways, and enteric viruses such as enteroviruses and rotavirus require infection of the intestinal epithelial cells (IECs) lining the gastrointestinal (GI) tract (see Figure 1 for a schematic of IECs). The polarized epithelium of both the respiratory and gastrointestinal systems is adept at restricting the movement of solutes, ions, macromolecules, and pathogens across the mucosa. This regulation can be attributed to the junctional complex between adjacent cells [composed of the tight junction (TJ), adherens junction (AJ), and desmosomes (Figure 1)] and to an intricate association of actin filaments that provides support to the subapical membrane and junctional complex. Cortical actin filaments directly below the apical cell membrane form a multifaceted network that provides a physical barrier to the penetration of viruses and endocytic vesicles. This network must be modulated not only for a virus to gain entry from the apical surface into the cell cytoplasm, but also to assist traffic from the cell periphery to deep within the cell. Thus, it is not surprising that many viruses have evolved highly varied strategies to dissolve or modulate the cortical actin meshwork to facilitate cell entry and/or trafficking. Because this alteration often occurs very early in the virus life cycle (generally well before the release of genomic content and replication), the actin cytoskeleton is often one of the first components of the host cell disrupted to facilitate virus entry. In this review, we will discuss the general cell biological properties of the actin cytoskeleton in polarized epithelial cells and how this network is manipulated during infection by a diverse array of viral pathogens. 

## 2. The Apical Actin Network

Polarized epithelial cells are characterized by the separation of distinct apical and basolateral domains that sort, traffic, and localize unique subsets of plasma membrane proteins. Epithelial cells undergo polarization through pivotal interactions with the actin cytoskeleton and associated signaling molecules, resulting in the formation of junctional complexes (described in detail below). For comprehensive reviews on the role of actin in epithelial cell polarization, see [1,2,3]. Actin, a 42-kDa component of the eukaryotic cytoskeleton, exists throughout the cell as either monomeric globular G-actin or polymeric filamentous F-actin. Actin participates in various cellular processes, including polarization, endocytosis, vesicle trafficking, signaling, adhesion, structural maintenance, migration, and division.

The apical plasma membrane surfaces of polarized epithelial cells are heavily associated with the actin cytoskeletal network. In the case of absorptive intestinal epithelia, the apical cell surface (lying closest to the lumen) is composed of finger-like projections called microvilli. Microvilli are comprised of parallel actin bundles that anchor to the subapical actin network through direct interactions with several actin bundling proteins. The ERM (ezrin, radixin, and moesin) family of actin-binding proteins provides stability to the microvilli and are important components of epithelial cell architecture as they provide a link between the cortical membrane and actin cytoskeleton. Although ezrin is not required for the formation of microvilli, it is critical for the association between the apical terminal actin web and the actin bundles comprising the microvilli [4]. 

Likewise, ezrin localizes to the apical domains of polarized airway cells, and may function to stabilize the association between cilia and the subapical actin web [5,6]. The apical surfaces of polarized airway epithelial cells are comprised of cilia that function in the clearance of pathogens, toxins, debris, and allergens from the upper airway surfaces [7,8]. Cilia are microtubule-based structures that anchor into the apical surface of the epithelial cell at microtubule-organizing centers known as basal bodies. The arrangement of the axoneme, or the ciliary microtubule core, may vary across cilia; however, all axoneme have nine tangential microtubule doublets. The axonemes of motile cilia have an additional inner doublet, and therefore are termed as having a 9 + 2 microtubule arrangement; whereas primary cilia lack this and are referred to as having a 9 + 0 arrangement [5]. Cilia also play vital roles as extracellular mechanosensors, as environmental sensors, and as sites of signal transduction, particularly during embryonic development. Therefore, viral pathogens that encounter the polarized epithelium are likely faced with the formidable task of dissociating the apical actin network and perhaps altering the structure and/or function of microvilli or cilia in order to gain access to the cell cytoplasm. 

## 3. The Tight Junction

It is currently accepted that the junctional complex between polarized epithelial cells is actually an intricate and highly regulated structure that actively participates in maintaining epithelial cell homeostasis. Tight junctions (TJs) are characteristically located at the apicolateral borders of adjacent epithelial cells, and form belt-like areas responsible for selectively regulating the passage of ions and neutral molecules through the paracellular space. Farquhar and Palade first identified the TJ in 1963 as the apical-most component of a tripartite junctional complex between neighboring cells [6]. Since this milestone finding, the increasing complexity of TJ organization and structure is reflected in the abundant protein components and the discovery of several families of integral membrane proteins that localize to this dense plaque region (for a complete review of these components, see [9,10]). For further explanation of other components of polarized junctional complexes (such as AJ and desmosomes), see [11].

Cell-cell junctions of both vertebrate and invertebrates are associated with a cytosolic plaque region that is enriched in multi-domain scaffolding proteins (reviewed in [12]). It remains unclear whether TJ plaque proteins directly determine the subcellular localization and structural organization of TJ transmembrane proteins, or whether their primary function is the recruitment of cytosolic regulatory proteins and signaling molecules to cell junctions. Based on the identification of a large number of cytoplasmic TJ plaque proteins and associated signaling molecules, the TJ emerges as a complex platform that is involved in the coordination of a vast array of cellular processes. Although TJ plaque proteins likely play a central role in stabilizing the junction, by directly and/or indirectly interacting with the actin cytoskeleton and forming multiprotein signaling complexes, another determinant of TJ structure and function relates to the composition of associated integral membrane proteins within the lateral membrane. 

The TJ is closely associated with the actin cytoskeleton and even subtle modulation of the cortical actin cytoskeleton can induce structural changes to the TJ. This strong association is attributed to the direct contacts between several TJ-localized protein components and actin. For example, the zonula occludens (ZO) family of cytoplasmic proteins localize to the TJ and directly interact with actin filaments via their carboxy (ZO-1, ZO-2) or amino (ZO-3) termini [13,14,15,16]. Manipulation of the interaction between the perijunctional actomyosin network and ZO-1 leads to loss of junctional integrity and barrier function, highlighting the importance of these interactions in maintaining epithelial architecture [17,18]. Likewise, integral membrane proteins associated with the TJ (such as occludin) also interact with actin, thereby providing an additional level of stability [15]. 

Given that one of the functions of the TJ is to restrict pathogen access to the interstitium, it is a paradox that a number of viruses utilize TJ-localized integral membrane proteins as receptors. All coxsackie B viruses (CVBs) and some adenoviruses (AdVs), bind to the coxsackievirus and adenovirus receptor (CAR), an integral membrane protein with two extracellular immunoglobulin-like domains localizing to the TJ [19,20]. Reoviruses bind to junctional adhesion molecule (JAM), a TJ‑localized integral membrane protein [21] and hepatitis C virus (HCV) utilizes two TJ-associated multi-spanning integral membrane proteins—occludin and claudin-1 to facilitate its entry [22,23]. Although the reason for the propensity of these viruses to bind TJ-localized receptors remains unclear, it is attractive to speculate that the close association of many TJ-localized components and the actin cytoskeleton serves as a central target for disruption induced by virus-receptor interactions. 

## 4. Rho Family GTPases in Polarized Epithelia

The ability of cells to alter their shape or to become motile is tightly regulated by the actin cytoskeleton. The Rho family GTPases (Rho, Rac, and Cdc42) are pivotal components of actin cytoskeletal regulation and when activated, signal to a variety of downstream effector molecules that govern coordinated and directed cellular movements. Rho, Rac, and Cdc42 regulate independent pathways to assemble distinct filamentous actin structures. Rho regulates the assembly of contractile actin-myosin filament bundles to form stress fibers that provide the driving force for cell movement; Rac mediates the formation of focal complex structures associated with lamellipodia/membrane ruffles; and Cdc42 activation promotes the formation of fingerlike structures of actin filaments known as filopodia. Rho family-mediated actin rearrangements require the polymerization of the actin cytoskeleton to coordinate cellular movements. Actin polymerization occurs by addition of actin monomers to the free barbed ends of an actin filament and depends on the activity of actin nucleating factors. For a complete review of the Rho GTPases and their roles in modulating actin in fundamental cellular processes, see [24,25,26,27]. 

It is well established that the actin cytoskeleton plays a critical role in maintaining TJ architecture and that perturbation of the actin cytoskeleton leads to drastic loss of TJ function. Depolymerization of the actin cytoskeleton by treatment of cells with actin-depolymerizing agents, such as latrunculin A (LatA), coincides with loss of barrier function and rapid internalization of junction-associated molecules, including occludin, ZO-1, and claudin-4 [28]. Both Rho GTPases Cdc42 and Rac exhibit pronounced association with the junctional complex, and thus, have been suggested to be direct regulators (further reviewed in [29]). Consistent with this, Cdc42 and Rac bind to the ASIP/Par-3 and Par-6/aPKC (atypical protein kinase C) complex localized to the TJ [30,31]. Par-3 is necessary for TJ assembly, and the establishment of epithelial cell polarity [30]. Although Par-6/aPKC is not essential for the development of TJ [30], the interaction between Par-3 and Par-6/aPKC is critical for the development of the apical domain during polarization [32]. For further review on the role of Rho GTPases in epithelial polarization, see [33,34]. 

## 5. Viral Manipulation of the Actin Network in Polarized Epithelial Cells

Despite the vast array of viruses that target a polarized epithelial monolayer for host invasion, relatively little is known regarding the precise mechanisms they utilize to alter the properties of these specialized cells in order to facilitate their entry, replication, or egress. This is likely attributed to the inherent complexity that might be expected to accompany virus entry into, or infection of, polarized cells. Below we summarize the mechanisms utilized by several viruses to disrupt the actin cytoskeleton to facilitate their entry and/or infection. We focus on viruses that are predicted to specifically target polarized cells at some stage of the virus life cycle, and on those whose entry pathways have been studied in polarized monolayers. In some cases, we highlight the mechanisms that these viruses utilize to modulate the actin cytoskeleton in non-polarized cells as a model for the changes that may occur in polarized cells. As this review is mainly limited to the role of actin in virus entry in polarized epithelia, for further discussion on the role of viral manipulation of the host actin cytoskeletal network throughout the entire virus life cycle in both non-polarized and polarized cells, see [35,36,37,38,39]. 

### 5.1. Rotavirus

Rotavirus, a non-enveloped, dsRNA virus from the *Reoviridae* family, is the most common viral causative agent of severe gastroenteritis in young children worldwide and accounts for as many as 500,000 deaths annually [40]. Given its clinical manifestations, it is clear that rotavirus is particularly adept at infecting polarized enterocytes of the GI tract, and subsequently, elicits powerful effects by disrupting epithelial integrity and barrier function. Indeed, rotavirus infection of polarized IECs in culture induces marked loss of junctional integrity, disrupts the actin cytoskeleton, and correspondingly increases paracellular permeability [41,42]. The dramatic increases in epithelial permeability induced by rotavirus have been linked to the specific effects of a non-structural glycoprotein, NSP4, whose expression alone is sufficient to cause epithelial cell leakage and tight junction disruption [43], diarrhea in mice [44], membrane destabilization [45], and intracellular calcium release [46,47]. Somewhat surprisingly, these actions are not sufficient to induce epithelial cell death and lysis, as rotavirus has been speculated to exhibit polarized release from the apical surface of enterocytes, presumably via a defined exocytic mechanism [48]. 

An association between rotavirus infection and rearrangement of the actin cytoskeleton has been linked to the specific effects of two rotavirus proteins—the aforementioned enterotoxin NSP4 and the capsid spike protein VP4. Expression of NSP4 is sufficient to induce profound rearrangements of the actin network in human embryonic kidney (HEK) cells and corresponding alterations in the phosphorylation state of the actin-binding protein cofilin [49]. In a parallel manner, VP4 interacts with actin bundles at the apical brush border of intestinal epithelia and induces rearrangements of the subapical actin network [50]. The propensity of two rotavirus proteins to interact with and disrupt the actin cytoskeleton points to the importance of this network in mediating enterocyte cell architecture and stability, and likely provides insights into the mechanisms by which rotavirus elicits such severe effects within the GI tract. 

### 5.2. Adenovirus

Adenoviruses (AdV) are non-enveloped icosahedral viruses composed of a nucleocapsid and a linear dsDNA genome. Pathologically, AdV are associated with acute respiratory infections in children, infections of the gastrointestinal tract, and viral conjunctivitis. The steps associated with the entry of several AdV serotypes have highlighted the complex interplay between these viruses and the host actin cytoskeleton [51,52,53,54]. Recently, a novel paradigm of virus entry has been identified in polarized airway epithelia using human adenovirus type 5 (Ad5) [54]. In a resting state, the Ad5 co-receptor αvβ3 integrin localizes to the basolateral domain in polarized airway epithelia. Thus, infection from the apical domain occurs at extremely low levels. However, if cytokine CXCL8 (interleukin-8) is present in the media during Ad5 infection, either due to co-culturing with macrophages on transwell inserts or using conditioned medium, virus infection occurs with high efficiency at the apical surface. CXCL8 is a ligand for apical chemokine receptors CXCR1 and CXCR2, and is required for phosphorylation and activation of both Src kinases and the focal adhesion‑associated molecule paxillin via these receptors. This signal transduction cascade, results in the translocation of αvβ3 and CAR to the apical domain for virus binding and infection. This study presents an elegant mechanism by which Ad5 hijacks and exploits host antiviral defense signaling pathways to facilitate its entry into polarized cells. 

In non-polarized HeLa cells, Meier *et al.* revealed that adenovirus type 2 (Ad2) utilizes a clathrin‑dependent entry mechanism that coincides with virus-dependent activation of macropinocytosis. The authors concluded that Ad2-induced signaling at the host cell surface was responsible for the accompanied stimulation of macropinocytosis. This process required α_v_ integrins, protein kinase C (PKC), F-actin, and the amiloride-sensitive Na^+^/H^+^ pump for AdV endosome release and host infection [51]. Similarly, adenovirus serotype 3 (Ad3) also exploits macropinocytosis to enter non‑polarized cells [52]. However, macropinocytosis appeared to be the major pathway required for Ad3 entry [52], rather than a consequence of viral-stimulation of the host cell as previously shown for Ad2 [51]. Macropinocytosis-dependent Ad3-entry requires F-actin, PKC, Na^+^/H^+^ exchanger, and Rho GTPase Rac1, but not Cdc42 [52]. Serine/Threonine kinase Pak1 [p21 (Cdc42/Rac)-activated kinase 1] and its effector the C-terminal binding protein 1 of E1A (CtBP-1) were also essential for Ad3 entry by macropinocytosis [52]. The authors also demonstrated that a minor clathrin-dependent Ad3 entry pathway likely exists [52]. 

Furthermore, adenovirus serotype 35 (Ad35) is dependent on F-actin, PKC, Na^+^/H^+ ^pump exchangers, Pak1, CtBP1, and Rac1 in multiple non-polarized cell types (HeLa, human kidney HK-2 cells, and human embryonic lung fibroblasts) [53]. However, as these studies of Ad2, Ad3, and Ad35 entry were conducted in non-polarized cell types, it remains unclear if these same mechanisms exist in polarized epithelia. Given that AdV infections are associated with both respiratory and gastrointestinal pathologies, and because many AdV serotypes bind CAR, it is likely that these viruses will also exploit the actin cytoskeleton to facilitate entry into polarized epithelia. Additional work in this area will also shed light on whether other AdV serotypes require host cytokines to permit entry across polarized epithelia, such as with Ad5 [54].

### 5.3. Influenza Virus

Influenza virus is an enveloped negative-sense ssRNA virus that belongs to the *Orthomyxoviridae* family, and causes highly contagious acute respiratory tract infections [55]. Interactions between influenza and the actin cytoskeleton have been cited throughout various phases of the virus life cycle, including entry, antiviral signaling, intracellular trafficking, and egress. Early work demonstrated a preference for influenza virus to enter at the apical surface of polarized epithelial monolayers [56]. Subsequently, studies using the actin depolymerizing drug cytochalasin D (CytoD) suggested that successful influenza entry at the apical domain in polarized epithelium requires interactions with the actin cytoskeleton, and that CytoD interferes with pan endocytosis at the apical surface [57]. More recent work indicated that the requirement of actin in mediating influenza virus entry varies across non-polarized and polarized epithelial cell types [58]. During entry into non-polarized cells (e.g., BHK, CHO, MV1 Lu, HeLa), influenza virus interactions with the actin cytoskeleton do not appear to be required, as CytoD did not affect infection in these cell types [58]. However, actin depolymerization with CytoD and stabilization with jasplakinolide both inhibited influenza entry and infection across a panel of polarized epithelial cells (e.g., MDCK II, Caco-2, Calu-3) [58]. Furthermore, the actin motor protein myosin VI is required for influenza entry in polarized epithelium, but not in non-polarized cell types [58]. Consequently, the actin cytoskeleton is necessary to facilitate the internalization of influenza virus into polarized epithelial cells.

Following viral entry, host cells often modulate innate immune signaling in response to microbial invasion. Interestingly, the actin cytoskeleton mediates antiviral signaling in response to influenza A virus infection of macrophages, non-polarized phagocytic cells that participate in innate immunity across various tissues of the body [59]. Following virus infection, actin levels were upregulated, along with translocation of viral RNA recognition components to the mitochondria, including retinoic acid‑inducible protein I (RIG-I), tumor necrosis factor receptor-1 (TNFR1)-associated death domain protein (TRADD), tripartite motif protein 25 (TRIM25), and IKKε (inducible IκB kinase) [59]. Depolymerization of actin with CytoD in primary human macrophages resulted in decreased expression of antiviral signaling molecules interferon-β (IFN-β), interleukin-29 (IL-29), and tumor necrosis factor-α (TNF-α) following influenza virus infection [59]. It will be interesting to determine whether this phenomenon is specific to non-polarized immune cells, and/or if similar mechanisms occur between influenza virus, actin, and innate immune components in polarized epithelia. 

Intracellular trafficking of influenza virus after fusion and entry has been visualized with real-time fluorescence microscopy in non-polarized Chinese hamster ovary (CHO) cells [60]. The actin cytoskeleton has been implicated in the post-fusion movement of virus-containing endosomes to the cell interior, prior to perinuclear translocation. Treatment of cells with CytoD restricted intracellular viral movement, suggesting that actin is responsible for the transport of these virus-positive vesicles [60]. However, the authors did not exclude the possibility that actin may be involved in the post‑binding movement of virus across the cell surface. Future work in polarized epithelial cells will also shed light on the contribution of actin to influenza virus trafficking following entry, and may also function in similar or in different ways than the mechanisms described above.

The actin cytoskeleton has also been implicated in the egress and budding of mature virions following successful influenza virus replication in host cells. Influenza virus buds at the apical surface as either spherical or filamentous enveloped virions, both of which are approximately 100 nm in diameter ([61]; reviewed in [62]). The budding of filamentous influenza virions has been suggested to occur through interactions with the host actin cytoskeleton [62,63,64]. Early evidence in polarized Madin-Darby canine kidney (MDCK) epithelial cells using CytoD demonstrated a significant reduction in the amount of filamentous (but not spherical) influenza virions released following actin disruption [63]. Removal of drug and cell recovery for three hours resulted in a corresponding increase in filamentous virion release; although, a considerable decrease in the length of the virions was observed compared to the untreated control. This work suggests that actin is involved in the budding, and in possibly regulating the size of filamentous influenza virions. More recently, actin has been associated with maintaining the lipid raft scaffold of the host plasma membrane for incorporation into the maturing filamentous influenza virions during budding events in MDCK cells [62]. 

Further insight into the mechanisms of actin involvement in influenza virion assembly and budding in non-polarized HEK293 cells has revealed the requirement for Rab11, a small GTP-binding protein that participates in endocytic recycling pathways, and Rab11 family interacting protein 3 (FIP3), a membrane trafficking and actin regulating protein [64]. Depletion of cellular Rab11 or FIP3 by siRNA resulted in a loss of the formation of filamentous virions. Interestingly, Rab11 was also found to be required for the budding of spherical virions, and loss of Rab11 caused a 100-fold reduction in the titer of spherical virions. Scanning electron microscopy (SEM) of Rab11-depleted cells demonstrated an accumulation of virions along the cell surface, indicating a budding defect. However, as these studies were performed in non-polarized cells, it is unclear if similar cellular components also facilitate viral budding from the apical surface of infected epithelia. Clearly, the actin cytoskeleton plays a vital role in the lifecycle of influenza virus throughout various stages, including entry, replication, antiviral signaling, and budding, a phenomenon which is also likely to be paralleled in polarized airway epithelia. 

### 5.4. Herpesviruses

Herpes simplex virus (HSV) is an enveloped dsDNA virus, and a member of the alpha-herpesvirus subfamily of *Herpesviridae*. Herpes-simplex virus type 1 (HSV-1) causes predominately oral mucosal lesions and/or encephalitis and lifelong latent infections in host neurons [55]. HSV-1 may also enter the corneal epithelium of the eye to induce a wide range of ocular diseases within the cornea and the surrounding trabecular meshwork [65]. Characteristic of many enveloped viruses, HSV-1 enters permissive cell types either by fusion at the host plasma membrane [66,67,68,69] or by an endocytic route [70,71,72,73]. 

In addition, evidence exists for the utilization of a phagocytic-like mechanism for HSV-1 entry in both non-polarized and polarized epithelial cell types [70,71]. In both of these studies, HSV-1 entry required the receptor nectin-1, a member of the immunoglobulin superfamily. The HSV-1 entry process also appears to be somewhat cell type specific. In certain non-polarized cell types, such as primary human corneal fibroblasts and CHO cells stably expressing nectin-1, HSV-1 entry occurs through a “phagocytosis-like” mechanism, including filopodia-like actin rearrangements and RhoA GTPase activation [71]. Clement *et al.* also demonstrated that actin depolymerizing agents CytoD and latrunculin B (LatB) are potent inhibitors of HSV-1 entry in these cell types. Further studies in polarized retinal pigment epithelial (RPE) cells, which have a natural tropism for HSV-1 infection, also reveal the role of actin in facilitating virus entry [70]. Prior to entry, HSV-1 virions are observed (by live cell imaging) to attach, and then to induce filopodia-like protrusions along the RPE cell surface. Actin depolymerizing agents CytoD and latrunculin A (LatA) also prevent HSV-1 entry in RPE. For a more extensive review of HSV-1 entry, signaling, and its effects on the host cytoskeleton in polarized and non-polarized cell types, see other articles published in this special issue [74,75,76,77]. 

The role of actin in mediating HSV-1 entry in both non-polarized and polarized epithelial cells exposes the skillful nature of viruses to exploit key host machinery to gain access to the cell. As further studies are conducted, it will be interesting to determine if HSV-1 enters non-polarized and polarized cell types similarly, or if the endocytic mechanisms differ between cell types. 

### 5.5. Coxsackievirus B

Coxsackievirus B (CVB) is a non-enveloped positive-sense, ssRNA enterovirus from the *Picornaviridae* family, and is the causative agent of viral pancreatitis, myocarditis, and aseptic meningitis [78,79]. Non-polio enteroviruses (EVs), including CVB, cause an estimated 10–15 million symptomatic infections in the United States each year [80]. However, the majority of CVB infections are asymptomatic and much less severe. CVB and other EVs are spread via the fecal-oral route. Thus, these viruses primarily enter the polarized epithelial cells of the gastrointestinal tract early in infection. 

All six CVB serotypes (CVB1-6) utilize TJ-associated type I transmembrane protein CAR (coxsackievirus and adenovirus receptor) to enter and infect cells [19,81]. Specific serotypes of CVB (CVB1, CVB3-RD, CVB5) interact with an additional attachment receptor—decay-accelerating factor (DAF, also known as CD55), a GPI-anchored membrane protein that localizes to the apical surface of polarized epithelia [82,83,84,85,86]. Although DAF-binding CVBs initiate their entry into polarized epithelia by first attaching to the apical cell surface, they must interact with CAR, localized at paracellular TJs, in order to initiate uncoating events necessary for infection. CVB3-RD accomplishes this task by exploiting the inherent signaling capacity of apically localized DAF. Despite it being anchored to the outer leaflet of the plasma membrane via a GPI anchor (and thus does not contain an intracellular domain), DAF and other GPI-anchored membrane proteins can be induced to form larger raft patches upon lateral crosslinking (most commonly with antibodies) [87]. Several families of signaling molecules are also enriched in lipid raft microdomains and may become activated upon clustering. CVB3-RD-induced DAF clustering at the apical surface activates Abl, a tyrosine kinase, which causes Rac-dependent actin reorganization and subsequent movement of CVB-DAF complexes to the TJ by an unknown actin-dependent process [82]. CVB relocalization to the TJ allows for virus-CAR interactions that are required to initiate subsequent entry events [82,88]. In polarized cells, CAR remains at the TJ and does not internalize with CVB, but CVB entry does modulate epithelial barrier function as evidenced by enhancement of ion and solute flux across the paracellular space [82,89]. This is divergent from the mechanism described for CVB entry in non-polarized HeLa cells, which induces the internalization of CAR [90]. Furthermore, endocytosis of the TJ protein occludin in macropinosomes is also required for CVB entry in polarized intestinal epithelial cells, and virus fails to internalize in the absence of occludin, although the precise role of occludin in facilitating virus entry is unknown [89]. An assortment of additional signaling molecules, including Rab34, Rab5, and Ras (small GTPases involved in membrane ruffling and macropinocytosis), are also required for CVB entry in polarized intestinal epithelia [89]. 

Thus, CVB entry into polarized intestinal cells is a complex process that requires the participation of several actin-associated signaling components and regulatory molecules. CVB exploits two key TJ molecules—CAR and occludin—and various small GTPases associated with the induction of actin‑dependent macropinocytosis to facilitate its entry into the polarized epithelium of the gastrointestinal tract. The targeting of crucial mediators involved in the maintenance of epithelial barrier function further supports the significance of the role of the actin cytoskeleton in protecting host cells from pathogen invasion.

## 6. Concluding Remarks

Although this review briefly discusses interactions between the actin cytoskeleton in polarized epithelial cells with several diverse viruses, there remains much to be discovered regarding the mechanisms employed by viruses to enter and infect polarized epithelia. Despite this, many studies, representing a range of viruses with tropism towards polarized epithelium, suggest that modulation of the actin cytoskeleton is likely central to this process. In addition, the propensity of disparate viruses to specifically target key members of the epithelial junctional complex suggest that this cellular structure may represent a strategic site exploited by viruses to weaken and/or disrupt actin filaments in order to facilitate their entry. Future work is sure to provide exciting new insights into the mechanisms at play during virus entry into and infection of polarized cells that will undoubtedly point to a prominent role for the actin cytoskeleton in these processes. 

## Conflict of Interest

The authors declare no conflict of interest.
